# Susceptibility to Pitting and Environmentally Assisted Cracking of 17-4PH Martensitic Stainless Steel Produced by Laser Beam Melting

**DOI:** 10.3390/ma15207121

**Published:** 2022-10-13

**Authors:** Nizar Guennouni, Daniel Maisonnette, Christophe Grosjean, Dominique Poquillon, Christine Blanc

**Affiliations:** 1 CIRIMAT, Université de Toulouse, CNRS, INP-ENSIACET 4 allée Emile Monso, CS 44362, CEDEX 4, 31030 Toulouse, France; 2 Pôle MMS, CETIM, 7 rue de la Presse, 42000 Saint-Etienne, France

**Keywords:** martensitic stainless steel, austenite, environmentally assisted cracking, pitting, pit initiation, pit propagation, chloride

## Abstract

Materials produced by additive manufacturing (AM) often have different microstructures from those obtained using conventional metallurgy (CM), which can have significant impacts on the materials’ durability, and in particular, resistance to corrosion. In this study, we were concerned with the susceptibility to pitting and environmentally assisted cracking (EAC) of 17-4PH martensitic stainless steel (MSS). We focused on the evolution from pitting to EAC, and the behaviour of MSS produced by AM was compared with that of its CM counterpart. Potentiodynamic polarisation tests were combined with chronoamperometry measurements performed without and with mechanical loading to study both stable and metastable pitting and the influence of stress on these processes. EAC tests were carried out and combined with observations of fracture surfaces. MSS produced by AM was more resistant to pit initiation due to fewer and finer NbC particles. However, the propagation kinetics of stable pits were higher for this MSS due to a higher amount of reversed austenite. The stress was found to stabilise the metastable pits and to accelerate the propagation of stable pits, which resulted in an increased susceptibility to EAC of the MSS produced by AM. These results clearly highlighted the fact that the reversed austenite amount has to be perfectly controlled in AM processes.

## 1. Introduction

Even when it comes to stainless steels, the problem of environmentally assisted cracking (EAC) arises. This is true for 17-4PH martensitic stainless steel (MSS) elaborated by conventional metallurgy (CM) whose susceptibility to EAC has been largely demonstrated in the literature, even if the studies have shown the difficulty of deconvoluting the role of hydrogen from that of the anodic dissolution on the damage [1,2,3,4]. With respect to all the other metallic materials, EAC is based on a microstructure—environment—stress state coupling [5]. Thus, it is relevant, when developing a new process for the elaboration of metallic materials, to determine how the microstructures generated by the process differ from those obtained by conventional metallurgy, and if the differences in the microstructures, even minimal, can lead to a significant modification of the material susceptibility to EAC. This is the question raised in the present study for 17-4PH MSS, which was developed using laser powder bed fusion (L-PBF). 17-4PH MSS is used in a wide range of applications (aerospace, energy, chemical industry, tooling, etc.) combining high requirements in terms of corrosion and mechanical properties. The diversity of ageing treatments that can be applied also makes it possible to adjust the strength/toughness compromise to the needs of the application. The low carbon content of 17-4PH MSS makes it a natural candidate for laser beam melting, unlike most high strength steels whose carbon content reduces their weldability, and therefore, makes them very difficult to use by this process.

Recently, there have been many studies in the literature on the microstructures of precipitation-hardening stainless steels generated by additive manufacturing (AM) processes, and in particular laser melting processes [6,7,8,9,10,11,12,13,14]. Most of the studies have highlighted differences in the austenite to martensite ratio in additive manufactured steels as compared with their conventional counterparts [9,10,11,12,13,14], in agreement with previous works showing variations of this ratio in a very large range up to 100% [15,16,17,18,19]. The results showed that the austenite to martensite ratio strongly depended on the chemical composition and microstructure of the powder used, as well as on the nature of the vector gas used during the AM process [15,19,20,21]. In particular, we have shown, in a previous work, that the microstructure of L-PBF 17-4PH MSS in the H900 metallurgical state (annealing at 1040 °C for 30 min, air-quench, ageing treatment at 482 °C for 1 h) was mainly martensitic, but with a non-negligible austenite content of about 13%, whereas the conventional counterpart only contained less than 1% austenite [13]. Here, it was interesting to note that post-building heat treatments, for example, H900, homogenised the microstructure (elimination of the dendritic solidification structure and crystallographic texture), whereas as-built parts were characterised by strong anisotropy in grain morphology and texture [14,16,17,18]. Furthermore, internal stresses have been generated during the building step [22,23] that could influence the repassivation ability of the steels [23]. Furthermore, they could also impact crack initiation and growth. Indeed, fatigue tests have been carried out for 17-4PH MSS in order to understand the influence of residual stresses on crack initiation [24,25,26]. For most of them, residual stresses were due to surface treatments that modified the surface roughness and induced microstructural and hardening gradients. The results have shown that a decrease in the surface tensile stresses delayed crack initiation [24,25]. The detrimental effect of residual stresses has also been shown during crack propagation tests on single edge-notched tension samples [26]. However, in a previous work, we showed that H900 heat treatment could efficiently release residual stresses [13]. Then, another issue of AM processes was the presence of gas pores and lack-of-fusion pores that depended on process parameters, but also powder choice, and could influence the corrosion resistance [16,18,27,28,29,30]. Similarly, Si-rich oxide inclusions have been considered to be manufacturing defects that could weaken the repassivation ability of the AM steels [31]. However, in our previous work, the porosity rate of the H900 AM samples was only 0.04% [13], in agreement with other works showing that this microstructural parameter could be controlled by a relevant choice of process parameter [16,23]. Barroux et al. worked on the same samples and confirmed that the pores and Si-rich oxide inclusions did not affect the pitting corrosion behaviour, whereas they showed that the austenite to martensite ratio was a first-order parameter in the pitting corrosion behaviour of 17-4PH MSS [32], in agreement with Tavakoli Shoushtari’s work [33]. This could be, at least partially, explained by referring to the influence of this ratio on the passive film properties [34].

Calabrese et al. [35,36] monitored the stress-corrosion cracking (SCC) process for 17-4PH MSS in a hot MgCl_2_ solution by electrochemical noise and acoustic emission techniques; they showed that the corrosion mechanisms evolved from localised pitting to SCC. This was in good agreement with largely accepted conclusions that assumed pits were preferential sites for SCC initiation for SS [37,38,39,40,41,42]. Therefore, it could be assumed that differences in the austenite to martensite ratio, which have been related to various pitting behaviours, were likely to lead to changes in the EAC susceptibility of 17-4PH MSS; thus, differences in EAC susceptibility between L-PBF MSS and its conventional counterpart could be observed. Furthermore, the film-rupture depassivation-repassivation (FRDR) model clearly correlated the rupture of the passive film due to mechanical loading and the EAC susceptibility [43,44,45,46,47]. In that sense, the passive film properties constituted a major parameter to explain EAC susceptibility; therefore, any microstructural changes leading to modifications in the passive film properties could lead to an evolution in the EAC susceptibility.

In this framework, the aim of the present work was to analyse the EAC susceptibility of L-PBF 17-4PH MSS as compared with its conventional counterpart. The microstructures of both AM and CM samples of MSS have been studied in detail in a previous paper [13]; only the main features were recalled in the present paper. The pitting corrosion behaviours of both AM and CM samples of MSS were first studied in a chloride solution. Then, their EAC behaviours were evaluated by performing interrupted EAC tests in the same solution. During exposure to chloride solution under a controlled strain, some samples were polarised in the passivity range; chronoamperometry measurements were performed, and the current transients were statistically analysed by comparing with the results obtained without mechanical loading. After the EAC tests, the samples were submitted to a tensile test; the corrosion damage on the gauge length and the fracture surfaces were analysed.

## 2. Materials and Methods

**Figure 1:**
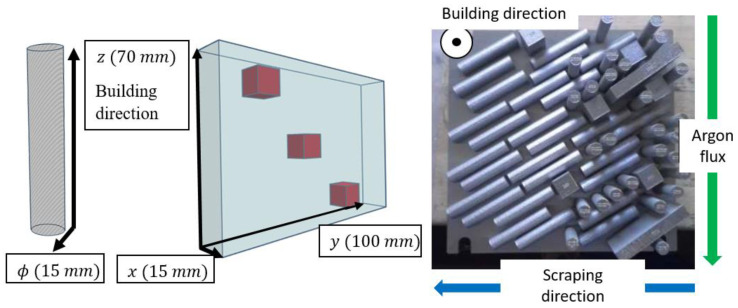
3D representation of the AM specimens and a photograph of the building plate with the built parts. Cylinders and parallelepipeds were built. Cubic samples were machined from the parallelepipeds after building.

The parts of 17-4PH MSS manufactured using L-PBF, here, referred to as AM, were built using an EOS DMLS M290 machine (400 W laser) under Ar, with the building plate maintained at 200 °C, a 40 µm layer thickness, and a 67° rotation between each layer. To select the process parameters, we performed a comparison with 5 manufacturers with their own parameters. On this basis, one manufacturer was selected for its metallurgical quality (few defects) of built parts and its traceability. In particular, the porosity rate was only 0.04%. Parallelepipeds (100 mm × 15 mm × 70 mm) and cylinders (15 mm in diameter and 70 mm in height) were built; the direction relative to the 70 mm side of the parallelepipeds and the cylinder axis was parallel to the building direction (Figure 1). Cubic samples were extracted from the parallelepipeds for the corrosion tests, while samples for EAC tests were machined using the cylindrical parts. For the conventional 17-4PH MSS, cubic samples and cylindrical samples (15 mm diameter) were extracted from a 50 mm diameter wrought cylinder for the corrosion and EAC tests, respectively, and were referred to as the CM samples. The chemical compositions of both the AM and the CM samples are presented in Table 1. The AM and CM samples were both studied in the H900 metallurgical state, as previously defined. As indicated in the Introduction section, this post-building heat treatment released the residual stresses due to the building process for the AM samples.

The scanning transmission electron microscopy (STEM) observations showed similar dislocation density for the AM and CM samples of MSS, probably due to the annealing treatment at 1040 °C. Energy-dispersive X-ray diffraction analyses with iterative peak simulations were performed to evaluate the austenite contents, which were 12.6 ± 0.3% and 0.8 ± 0.2% for the AM and CM samples, respectively. For the AM samples, the main component of the austenite was reversed austenite, as shown by the Ni maps obtained by using STEM energy dispersive spectroscopy. The width of the martensite laths was between 50 and 800 nm; this fine martensitic microstructure had probably contributed to promoting the nucleation and growth of the reversed austenite which was mainly found at the martensite lath boundaries. The AM samples contained NbC precipitates with a mean diameter of 0.10 ± 0.01 µm, and Cu precipitates with a diameter varying from 10 to 15 nm. The CM samples had a fully martensitic microstructure. The width of the martensite laths was between 700 nm and 2.5 µm. Three times more numerous NbC precipitates than in the AM samples were observed; they were larger, with a mean diameter of 0.13 ± 0.01 µm. On the contrary, finer Cu-rich precipitates (approximately 4 nm in diameter) were observed in the CM samples as compared with the AM samples.

**Figure 2:**
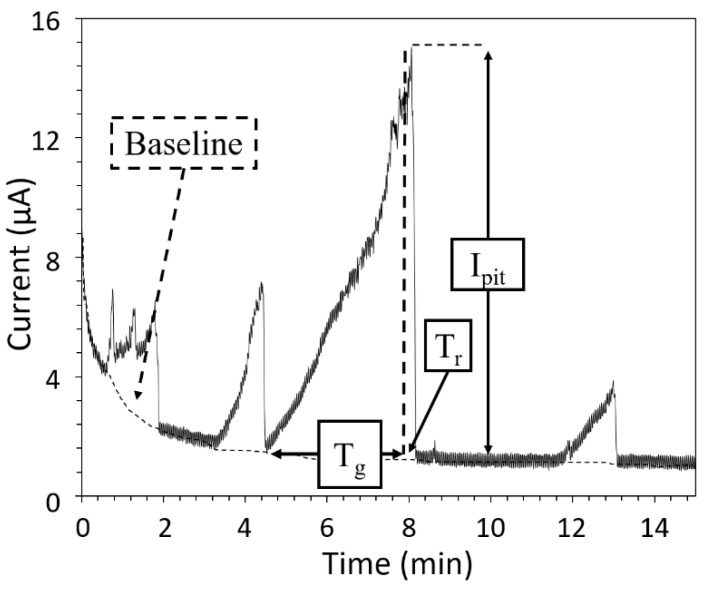
Example of a current transient measured during the potentiostatic experiments (samples maintained at a constant potential of 250 mV versus E_corr_ for 1 h). Identification of the values characteristic of the metastable pits, i.e., E_pit_, T_g_, and T_r_.

The corrosion tests consisted of potentiodynamic (polarisation curves) and potentiostatic (chronoamperometry) experiments performed using a VSP Biologic potentiostat, in a 0.5 M NaCl solution maintained at 25 °C, stirred, and open to air. Before all experiments, the samples were immersed at their open circuit potential (OCP) for 1 h in order to reach near steady state conditions. Cubic samples were used for both the AM and CM samples of MSSs. Their surfaces were mechanically ground down to 2400 grade with SiC paper, then, polished down to 1 µm with diamond paste, and rinsed with deionised water in an ultrasonic bath before the corrosion tests. The samples were mounted into an epoxy resin with a Cu wire for electric connexion; the surface exposed to the electrolyte was equal to 1 cm^2^. For the AM samples, the experiments were performed with the surface exposed to the electrolyte corresponding to a plane parallel or perpendicular to the building direction. Differences were observed in the corrosion behaviour depending on the orientation of the surface relative to the building direction. However, whatever the surface exposed, the conclusions were similar. Therefore, only the results obtained with a surface parallel to the building direction are given in the present manuscript for brevity reasons. For the CM samples, the surface exposed was perpendicular to the axis of the 50 mm diameter cylinder. For all experiments, the three-electrode cell was completed using a saturated calomel electrode (SCE) as a reference electrode and a platinum foil as a counter electrode. The polarisation curves were plotted from the OCP to −500 mV_SCE_ for the cathodic curves, and to a potential corresponding to a current value equal to 200 µA cm^−2^ for the anodic curves; the scan rate was 500 mV h^−1^. All curves were plotted at least three times for reproducibility. Two sets of chronoamperometry measurements were performed. First, the samples were maintained for 30 min at +500 mV versus the corrosion potential (E_corr_) to characterise stable pits; the current value was recorded every 0.1 s. At the end of the test, the morphology and sizes of the pits were determined by using a Zeiss Axiover A 1 M microscope; the samples’ surfaces and cross-sections were observed. Observations were completed using an S Neox Sensofar confocal and interferometric microscope, which allowed characteristic values of the pit morphology (depth and diameter) to be extracted from the mean profiles of the pits. For both the AM and CM samples, these experiments were reproduced four times in order to generate enough pits for the analysis. The other samples were maintained in the passivity range, at a constant potential of +250 mV versus E_corr_, in order to study the metastable pits. The current response was recorded every 0.1 s. These measurements were performed without and with mechanical loading. For the measurements with mechanical loading, they were performed in the cell designed for the EAC tests using a Voltalab PGP201 potentiostat. The samples were axisymmetric tensile specimens machined to 4 mm diameter and 34 mm gauge length from 15 mm diameter cylinders. They complied with the recommendations of the DIN EN ISO 6892-1 standard relevant for uniaxial tensile tests for metallic materials. The surface exposed to the electrolyte was prepared as previously described, using a specific setup; a silicone mask was used to expose only a 30 mm gauge length and to electrically isolate the tensile specimens from the testing machine (all the metallic parts of the testing machine were isolated from the electrolyte). Chronoamperometry measurements without mechanical loading were performed using two configurations: with axisymmetric tensile specimens in the EAC cell for a better comparison with the chronoamperometry tests with mechanical loading, and with cubic samples in a beaker as for the first set of chronoamperometry measurements. Threshold values of 50 and 100 nA (higher value due to the noise related to the electrolyte stirring in the EAC cell) above the background current were chosen to identify the current transients corresponding to the metastable pits for cubic samples and axisymmetric samples, respectively. For each configuration, the tests were replicated 5 times so that a statistical analysis of the current transients could be performed. Figure 2 shows an example of such a current transient, and also introduces how the data were analysed. Three characteristic values were extracted. The maximum value reached by the current transient before going down to the background current was called I_pit_. T_g_ and T_r_ were the times for the current to reach I_pit_, and to go down to the background current value, respectively. T_g_ and T_r_ characterised the growth and repassivation of a metastable pit, respectively. This analysis has been described with more details in [32].

In order to evaluate the mechanical properties of the AM and CM samples, preliminary tensile tests were performed up to fracture, in air, with non-exposed tensile specimens and at a 10^−3^ s^−1^ strain rate, using a MTS machine with a 30 kN force cell and an extensometer with a gauge length of 25 mm. The extensometer was centred in the middle of the specimen gauge length. This allowed the 0.2 offset yield strength (YS_0_._2_) to be determined.

For the EAC tests, the tensile specimens were exposed to 0.5 M NaCl electrolyte in a home-designed electrochemical cell mounted on the MTS tensile machine. The strain was measured using a linear variable differential transformer (LVDT) sensor. Upon immersion in the electrolyte, the tensile specimens were submitted to mechanical loading at an imposed strain rate of 10^−3^ s^−1^ until a strain equivalent to an initial stress of 80% of the YS_0_._2_ value was reached. During the mechanical loading step, the corrosion potential E_corr_ of each specimen was recorded based on OCP measurements. Then, the strain was maintained for 60 min, during which time the specimen was either left at OCP or left at OCP for 40 min, and then polarised at +250 mV relative to the E_corr_ (average of OCP values measured over the last few minutes) for 20 min. In the latter case, the EAC tests were completed using a chronoamperometry test during the polarisation period, as previously described. Then, a tensile test up to fracture was performed at the same strain rate of 10^−3^ s^−1^. For the first series of tests, this was done in the electrolyte, but the polarisation was stopped (for tests with polarisation). For the second series of tests, the cell was emptied of the electrolyte, the specimen was cleaned with distilled water, and submitted to the tensile test in air.

After all mechanical tests, the fracture surfaces and the gauge lengths were observed using scanning electron microscopy (SEM), and the diameters of the tensile specimens were optically measured along the gauge length.

Regardless of the experiments (chronoamperometry measurements, EAC tests, tensile tests), a new tensile specimen was used for each experiment. Therefore, the experiments were performed using a very large number of cylindrical specimens, considering that a given test was repeated at least three times for reproducibility reasons. The preliminary study was performed with 5 manufacturers which allowed us to optimise the building step in such a way as the results obtained using different cylindrical specimens were reproducible. Similarly, observations were performed to check the homogeneity in the microstructure of the 50 mm diameter wrought cylinder to ensure the validity of the results obtained using a new axisymmetric tensile specimen for each test.

## 3. Results

### 3.1. Corrosion Behaviour of the LBM and CM 17-4PH MSSs

#### 3.1.1. General Corrosion Behaviour

**Figure 3:**
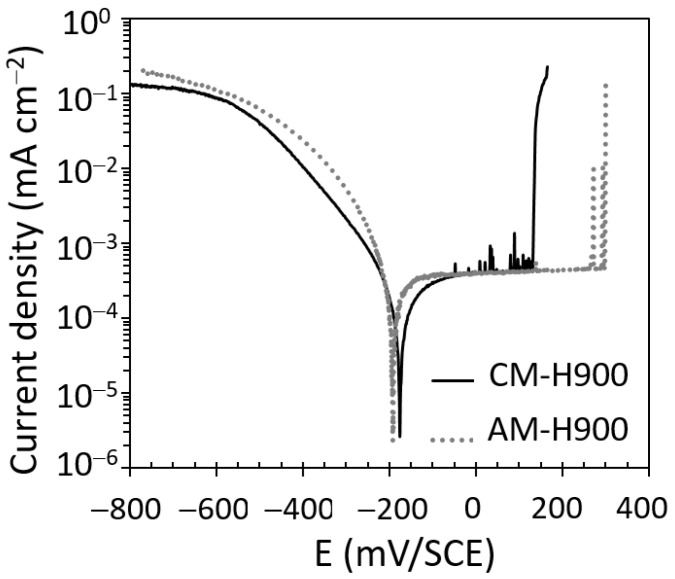
Polarisation curves plotted in 0.5 M NaCl solution for the AM and CM samples. Scan rate = 500 mV h^−1^.

The polarisation curves plotted for the AM and CM samples are shown in Figure 3. The cathodic branch corresponded to the oxygen reduction and showed similar current densities for both the AM and CM samples of MSS. Similar E_corr_ values were also obtained for the AM (−183 ± 4 mV_SCE_) and CM (−179 ± 1 mV_SCE_) samples of MSS. The anodic branch was first characterised by a very well-marked passivity plateau, as commonly observed for SS. The corrosion kinetics was, therefore, primarily controlled by the resistance of the passive film, so that the passive current density (i_pass_) was equal to the corrosion current density (i_corr_), with very similar values of i_pass_, and thus i_corr_, for both MSSs (0.33 ± 0.01 and 0.39 ± 0.02 µA cm^−2^ for the AM and CM samples of MSSs, respectively). Then, a breakdown potential was observed corresponding to an abrupt increase in the anodic current. The optical and SEM observations of the samples after the polarisation tests showed the formation of pits, and therefore, this breakdown potential was identified as the pitting potential E_pit_. The E_pit_ values were more positive for the AM samples (291 ± 9 mV_SCE_) than those for the CM samples (123 ± 11 mV_SCE_). This could be explained by referring to the distribution of NbC carbides (fewer and finer for the AM samples as compared with the CM samples), which constitute preferential sites for pit initiation [32,34]. Therefore, the results showed that MSS produced by AM had better resistance to pit initiation than MSS obtained by CM, in agreement with Barroux’s work [32,34]. Furthermore, current transients characteristic of metastable pits were observed for both MSSs; they were more numerous, but with a lower intensity, for the CM samples of MSS as compared with the AM samples of MSS.

#### 3.1.2. Susceptibility to Metastable Pitting

**Figure 4:**
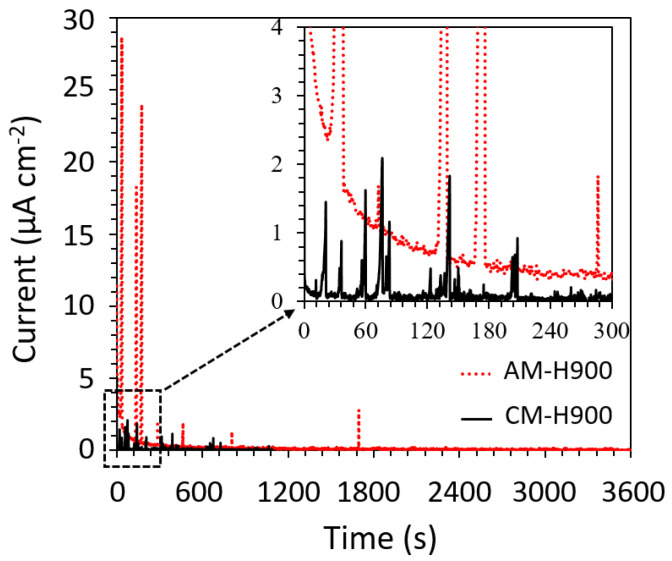
Current transients recorded during chronoamperometry tests performed in 0.5 M NaCl solution for the AM and CM samples at a fixed potential of +250 mV versus E_corr_.

To further evaluate the susceptibility to metastable pitting of the two MSSs, chronoamperometry tests were performed. Considering the width of the passivity plateau, the samples were polarised at a fixed potential equal to +250 mV relative to E_corr_. The I = f(t) curves representative of the AM and CM samples of MSS during chronoamperometry tests are presented in Figure 4. This figure shows current transients for both MSSs, but the transients seemed to be of lower intensity for the CM samples than for the AM samples, as observed in Figure 3, and in agreement with the work of Barroux et al. [32].

The current transients were counted and characterised by identifying the relevant characteristic parameters, as shown in Figure 2. By using average values of I_pit_ and T_g_, the growth kinetics of the current transients were also calculated through the ratio I_pit_/T_g_. The average values of these parameters are presented in Table 2. The statistical analysis of the current transients first confirmed that the current transients were more intense for the AM samples than for the CM samples, as suggested in Figure 4. However, these transients were more numerous (by a factor of 4) for the CM samples than for the AM samples. In addition, the T_g_ values were larger for the AM samples than for the CM samples, and the growth rate of metastable pits was significantly higher for the AM samples than for the CM samples (by a factor of 2.4). These results, therefore, showed that the susceptibility to metastable pitting could not be assessed on the basis of the number of current transients alone. Considering the other parameters, MSS produced by AM seemed to be more susceptible to metastable pitting than MSS obtained using CM.

The results obtained here were in perfect agreement with those obtained by Barroux et al. who also highlighted the greater susceptibility of AM samples to the formation of metastable pits as compared with CM samples [32]. The authors used XPS to analyse the chemical composition and thickness of the passive film formed on AM and CM samples of MSS [34]; they showed that the passive film was less rich in Cr for the AM samples than for the CM samples due to the higher content of reversed austenite in the AM sample, the austenite containing less Cr than the martensite. These conclusions were in good agreement with the work of Långberg et al. who compared the composition of the passive film above ferrite and martensite in a duplex SS [48]. This could explain the poorer repassivation capacity of the MSS produced by AM, and therefore, the higher I_pit_, T_g_, and growth kinetics, and even longer T_r_, for the AM samples than for the CM samples.

These differences in the behaviour of MSSs produced by AM and CM with respect to the formation of metastable pits raise questions about the susceptibility of the two steels to the initiation and propagation of stable pits, bearing in mind that it was shown in Figure 3 that the AM samples of MSS, with more positive E_pit_ values than the CM samples of MSS, were more resistant to the initiation of stable pits. Therefore, it was necessary to go further in this analysis by carrying out chronoamperometry tests for applied potentials more positive than E_pit_.

#### 3.1.3. Susceptibility to Stable Pitting

**Figure 5:**
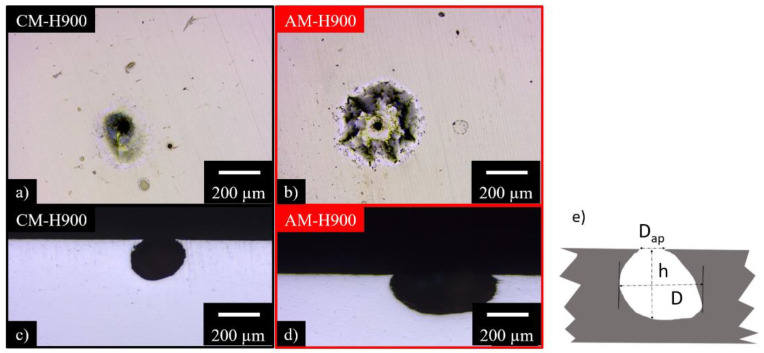
Optical microscope observations of the stable pits formed after chronoamperometry tests performed in 0.5 M NaCl solution at a fixed potential of +500 mV versus E_corr_ for: (**a**,**c**) CM samples; (**b**,**d**) AM samples; (**e**) scheme of a pit with the parameters used for the analysis.

In that case, chronoamperometry tests were performed at a potential corresponding to +500 mV relative to E_corr_, i.e., after E_pit_. The current density versus time measured during the tests (not shown) for both MSSs was almost twice as high for the AM as for the CM samples, whereas the ΔE = E − E_corr_ imposed for both materials was identical, and the E_pit_ values were more positive for the AM than for the CM samples. This would suggest that pits would propagate more rapidly in AM samples once initiated. Preliminary observations (Figure 5) of the pits showed that the morphology was similar between the two MSSs. However, the pits seemed to be larger for the AM than for the CM samples, which was consistent with the measured current densities.

A statistical analysis of the stable pits formed as a result of the tests was carried out to verify these observations. A confocal microscope was used to determine the pit depth, h, and cross-sections were studied to measure the pit diameters D and D_ap_, where D is the maximal pit diameter on the cross section view, and D_ap_ is the aperture diameter measured at the sample surface (Figure 5e). Finally, with these parameters, an average pit shape factor, f, given by the D/h ratio, was also estimated. The results of this statistical analysis are presented in Table 3. The quantitative analysis showed that the average number of stable pits per unit area was similar for the AM and CM samples of MSS, in agreement with the work of Barroux et al. [32]. However, contrary to what was shown by the latter authors, differences were observed in the present work with regard to the morphology of the pits, with notably larger pits, and even deeper, for the AM samples as compared with the CM samples. The result was consistent with the higher measured anodic current densities for the AM than for the CM samples. As mentioned earlier, pitting propagation seemed to be faster for the AM than for the CM samples. In view of the microstructural differences observed between the two MSSs, it was interesting to assess the role of reversed austenite. Thus, combining these results with those presented by Barroux et al. [32,34], in particular concerning the lower Cr amount of the passive film above the reversed austenite, the results showed that it was more difficult to initiate stable pits for MSS produced by AM because it contained fewer NbC particles, and the particles were smaller in size. However, once pitting had started, pits extended more rapidly in MSS produced by AM, particularly due to the lower Cr amount in the passive film, which made MSS produced by AM less able to repassivate. In addition, pits constituted a confined environment where acidification could occur rapidly due to corrosion reactions followed by hydrolysis of cations, and then the production of protons; this led to the absorption of hydrogen by the metal. The influence of hydrogen on MSS dissolution was not the focus of this paper, even though authors have shown a significant effect of hydrogen on the anodic processes [44]. Finally, it was interesting to note that the f values were very different between the AM and CM samples of MSS, due to the fact that the diameter (D) of the pits, on average, was twice as large as the depth (h) for MSS produced by AM. This suggested that the distribution of mechanical stresses should be different around the pits for the two MSSs under mechanical loading, which should influence their abilities to initiate cracks at the bottom of the pits during the EAC tests.

### 3.2. EAC Susceptibility of the LBM and CM 17-4PH MSSs

#### 3.2.1. General EAC Behaviour

**Figure 6:**
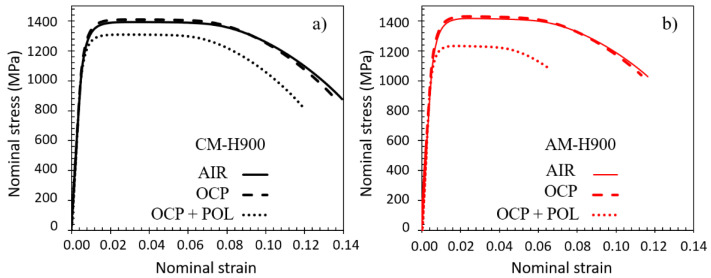
Nominal stress vs. nominal strain curves (10^−3^ s^−1^) obtained during the EAC tests for (**a**) CM and (**b**) AM samples for two experimental conditions: samples maintained at OCP and tests with a polarisation period (OCP + POL). For both MSSs, curves representative of their mechanical behaviours in air, without any exposure to NaCl solution, were reported (AIR) for comparison.

The nominal stress versus nominal strain curves plotted in NaCl solution for both AM and CM samples after the EAC tests are shown in Figure 6. The results obtained for samples left at the OCP and for those left at the OCP, and then polarised during the tests, are given. The curves plotted in air for the samples non-exposed to NaCl solution are also reported for comparison. During all tensile tests, necking was observed for both MSSs. The parameters that are characteristic of the mechanical properties were extracted from these curves, i.e., Young modulus E, yield strength YS_0_._2_, and ultimate tensile strength (UTS), and are reported in Table 4. Concerning the strain to fracture, ε_f_, the classical assumption of volume conservation during plastic deformation was made to take necking into consideration, i.e., to consider the heterogeneity in strain distribution all along the gauge length of the tensile specimens: ε_f_ = 2ln (ϕ_0_/ϕ_f_), with ϕ_0_ the initial diameter of the tensile specimen and ϕ_f_ the minimum diameter of the tensile specimen measured close to the fracture surface. This explained the differences between the strain to fracture values in Table 4 as compared with the curves from Figure 6. First, the results showed that the E, YS_0_._2_, and UTS values were similar for the non-exposed AM and CM samples [13,16]; lower values of ε_f_ were measured for the AM samples as compared with the CM samples, which could be due to the pores in the AM samples, even though the porosity rate was very low [13]. Then, after exposure to NaCl solution at the OCP, the mechanical responses of both MSSs were not significantly changed. On the contrary, for the samples polarised during 20 min, sharp decreases in YS_0_._2_, UTS, and ε_f_ values were observed, which were much more marked for the AM samples than for the CM samples, with a decrease of 10% versus 4% in YS_0_._2_ values and 12% versus 5% in UTS values, for the AM and CM samples, respectively. The decreases in ε_f_ values were similar for the two MSSs, i.e., 16% versus 14%, for the AM and CM samples, respectively. It was of interest to note that similar results were obtained when the tensile tests were performed in air after the exposure to NaCl solution under mechanical loading. This led us to assume that the period during which the samples were maintained under a constant strain in NaCl solution played a major role in modifying the mechanical behaviour of these MSSs.

**Figure 7:**
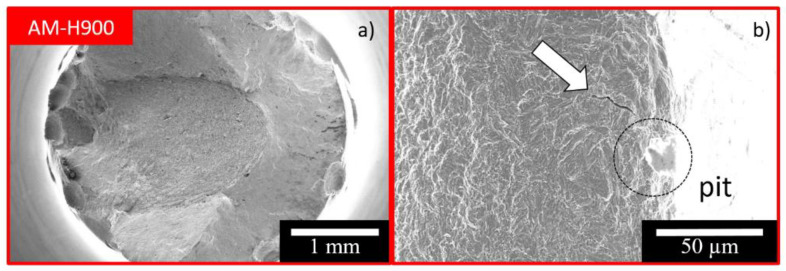
SEM observation of the fracture surface of an AM sample after an EAC test (sample maintained at the OCP and then polarised): (**a**) General view; (**b**) focus on a small crack initiated on pits (white arrow).

**Figure 8:**
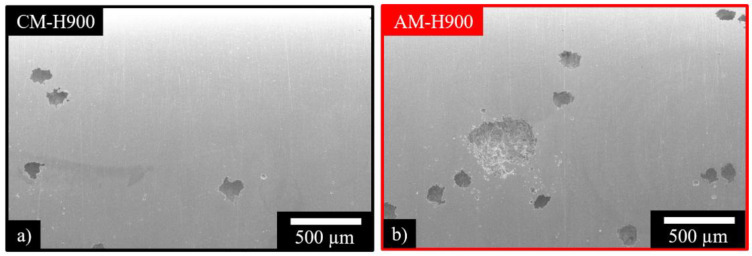
SEM observation of pits for: (**a**) CM; (**b**) AM samples. Pits were observed on the gauge length for samples polarised during the EAC tests.

For both MSSs, and for all the EAC tests, the fracture surfaces were globally ductile (Figure 7). For some specimens, which had been polarised during the EAC tests, pits were observed on the gauge length (Figure 8) even though the samples had been polarised at a potential which was situated in the passivity domain determined for the specimens which were not submitted to mechanical loading (Figure 3). Furthermore, small cracks were observed in a direction perpendicular to the mechanical loading axis (white arrow in Figure 7b)). Cracks were initiated at pits, which was in agreement with the commonly accepted hypothesis that pits act as preferential crack initiation sites [37,38,39,40,41,42,49].

#### 3.2.2. Stable Pitting Behaviour under Mechanical Loading

**Figure 9:**
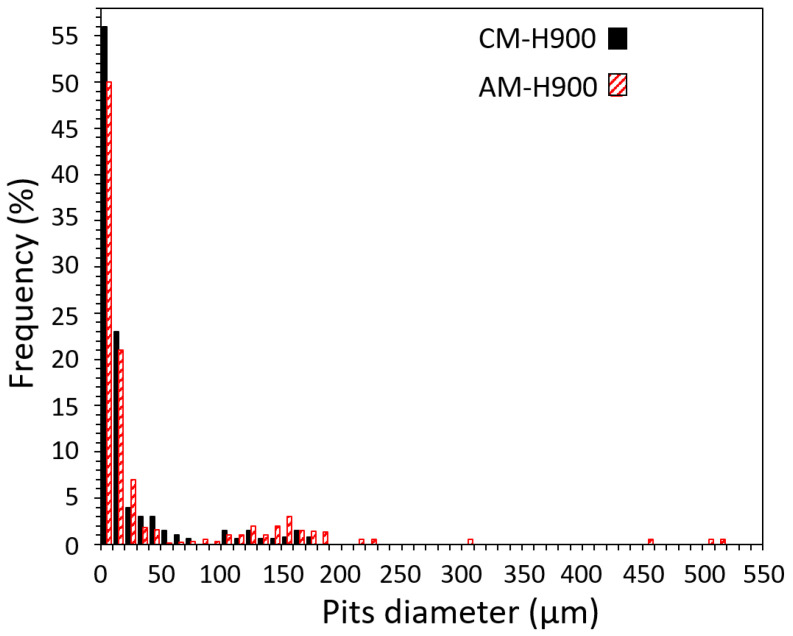
Statistical analysis performed on the stable pits observed for the AM and CM samples after some EAC tests in which the samples were polarised for 20 min during exposure to NaCl solution (total population of pits analysed: 420 and 480 for the AM and CM samples, respectively).

After some EAC tests, in which the samples were polarised at a potential corresponding to the passivity range of the MSSs when no mechanical loading was applied, a sharp and monotonic increase in current was recorded during the chronoamperometry measurements. This was associated with the formation of stable pits during the exposure to NaCl solution under mechanical loading, as observed previously (Figure 8). A statistical analysis of pit size was carried out by considering all the pits observed on the EAC samples in which pitting occurred. Approximately, 420 pits were observed for the AM samples and 480 pits were observed for the CM samples. For both MSSs, three populations of pits were identified depending on their sizes, but larger pits were globally observed for the AM samples as compared with the CM samples, as shown by the distribution of pit sizes (Figure 9).

The results clearly demonstrated that mechanical loading/environment coupling occurred during mechanical loading under controlled strain; this coupling effect resulted in the formation of pits at a potential in which passivity was observed when no mechanical loading was applied. Furthermore, the results clearly showed that the AM samples were more susceptible to this coupling effect than the CM samples, which could be related to the higher decrease in the mechanical properties observed for the AM samples after the EAC tests as compared with the CM samples (Figure 6 and Table 4). This confirmed that the pit to crack transition was a major phenomenon to explain the susceptibility to EAC of the 17-4PH MSS in those experimental conditions. It was also of interest to note that the observation of larger pits for the AM samples than for the CM samples following the EAC tests could be correlated with the previous chronoamperometry results obtained without mechanical loading, where pits were found to propagate more easily in the AM samples than in the CM samples (Figure 5 and Table 3). This led us to assume that the mechanical loading/environment coupling worsened the intrinsic susceptibility to pit propagation observed for the AM samples, which was explained, at least partially, by the amount of reversed austenite. Clearly, the influence of hydrogen on pit propagation could not be neglected, as mentioned previously. Furthermore, the influence of hydrogen on mechanical loss was also suspected. The hydrogen amount was measured using a melting method after the EAC tests under controlled strain rate (10^−3^ s^−1^) in NaCl solution: the hydrogen amount was about 3 (2.6) and 3.2 (3.2) ppm for the AM (CM) samples after tests performed at the OCP and under polarisation, respectively, whereas the hydrogen amount was about 1.5 ppm for the non-exposed samples.

#### 3.2.3. Metastable Pitting Behaviour under Mechanical Loading

**Figure 10:**
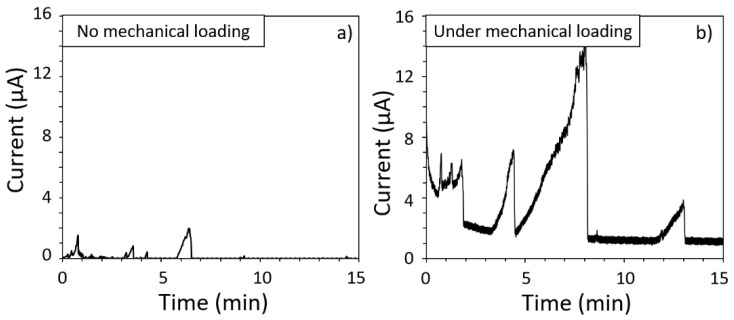
Current transients observed on samples exposed to 0.5 M NaCl solution: (**a**) Without mechanical loading; (**b**) with mechanical loading (EAC tests with an imposed strain corresponding to an initial stress of 80% of the YS_0_._2_ value). Results are given for the AM MSS samples.

Considering the results, attention was also given to the effect of mechanical loading on the susceptibility to metastable pitting of the MSSs. Indeed, during the EAC tests performed under polarisation, two sets of results were obtained. For some tests, stable pitting was observed during the chronoamperometry measurements, as previously discussed in Section 3.2.2. However, for some other tests, only metastable pitting was recorded, with similar current transients as those shown in Figure 2. In Figure 10, the current versus time measured on specimens exposed to 0.5 M NaCl solution in the EAC cell without mechanical loading (Figure 10a) is compared with mechanical loading, i.e., during the EAC tests (Figure 10b). The results are given for AM samples, but the conclusions were similar for the CM samples. Moreover, it was of interest to note that the results obtained without mechanical loading in the EAC cell were globally similar to those obtained with the cubic samples (Section 3.1.2). That said, Figure 10 clearly shows that the current transients were different for a test with mechanical loading as compared with a test without mechanical loading, with much more intense current transients under mechanical loading.

**Figure 11:**
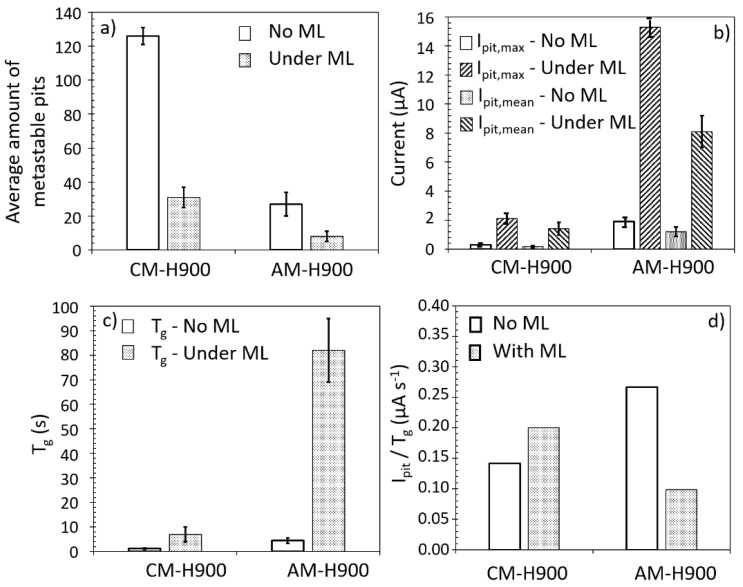
Results of the statistical analysis of the current transients observed for the samples exposed to 0.5 M NaCl solution, without mechanical loading (ML), and with mechanical loading (EAC tests with an imposed strain corresponding to an initial stress of 80% of the YS_0_._2_ value). The results are given for the AM and CM samples of MSS: (**a**) Average amount of metastable pits; (**b**) I_pit_ (max and mean values); (**c**) T_g_; (**d**) I_pit_/T_g_.

The results from the statistical analysis of these current transients are given in Figure 11 for both the AM and the CM samples of MSS. The number of current transients decreased sharply from 126 to 31 for the CM samples, and from 27 to 8 for the AM samples, when mechanical loading was applied (Figure 11a). However, the T_g_ values (Figure 11c), and also the T_r_ values (not shown), increased for both MSSs when mechanical loading was applied, which was more marked for the AM samples than for the CM samples. The results, therefore, showed that mechanical loading resulted in an increase in the average lifetime of the current transients, which was likely to be associated with an increase in the probability of stabilising these metastable pits. This was consistent with the observation of stable pits for some EAC tests. Furthermore, the highest influence of mechanical loading on metastable pits for the AM samples compared with the CM samples was in good agreement with the larger stable pits observed for the AM samples after the EAC tests. The increase in I_pit_ values (Figure 11b) with mechanical loading, which was also much more significant for the AM samples than for the CM samples, confirmed the conclusions. The very high increase in T_g_ values with mechanical loading as compared with the tests without mechanical loading led to a I_pit_/T_g_ ratio lower when mechanical loading was applied for the AM samples (Figure 11d).

The results, therefore, showed that the mechanical loading/environment coupling also promoted the transition from metastable to stable pitting, especially for the AM samples, in agreement with the stronger susceptibility to EAC of the AM samples as compared with the CM samples. This was relevant with EAC susceptibility mainly explained by the pit to crack transition, and could be explained by simply considering the effect of stress on the passive film properties, as proposed by different authors [50,51]. However, again, the role of hydrogen in the results obtained here could not be disregarded, i.e., the effect of mechanical loading on the hydrogen content inside the material, and the subsequent effect of hydrogen on the dissolution kinetics.

## 4. Discussion

The results clearly showed that the susceptibility to EAC of both the AM and CM samples of 17-4PH could be correlated with the susceptibility to pitting corrosion of those MSSs. The SEM observations of the fracture surfaces confirmed that anodic dissolution processes played a major role in the EAC susceptibility of these alloys, with in particular, pits acting as preferential sites for crack initiation (Figure 7). However, the tensile tests performed after exposure to NaCl solution showed greater EAC susceptibility of the AM samples as compared with the CM samples (Figure 6), whereas the AM samples were found to be more resistant to pit initiation than the CM samples, with significantly nobler E_pit_ values (Figure 3). This showed that the relationship between pitting and EAC susceptibility was not trivial; the pit to crack transition had to be considered, which implied the need to consider pit morphology in relation to stress distribution, and therefore, to include, in the analysis, both steps of the pit life, i.e., initiation and propagation. In that sense, the influence of microstructural features on the EAC susceptibility of 17-4PH MSS was rather complex, and the interest of AM processes in terms of durability of structural parts, in particular EAC susceptibility, has to be thoroughly evaluated. For the 17-4PH AM samples, the fewer and finer NbC particles as compared with the CM samples constituted a positive microstructural feature in terms of susceptibility to pit initiation, and then to EAC. However, the L-PBF process could also lead, which was the case with the experimental conditions fixed here, to a significantly higher amount of reversed austenite as compared with the conventional metallurgy [9,10,11,12,13,14,15,16,17,18,19]. The results showed that the high austenite amount was associated with a higher pit propagation rate (Figure 5 and Table 3), leading to significantly different pit shape factors (Table 3), as well as more intense metastable pits with a longer lifetime (Figure 4 and Table 2) for the AM samples as compared with the CM samples. This was explained by referring to literature data [32,34], which showed Cr impoverishment in the passive film formed on the reversed austenite as compared with the martensite. This was also in agreement with the recent work of Carvalho et al. [52] who analysed the susceptibility to pitting corrosion of a martensitic—ferritic SS, and showed that, for some microstructural states, pitting initiation took place mostly at Ni-enriched locations which were also Cr-depleted areas.

Furthermore, the issue was even more complex because the influence of mechanical loading on the microstructure/environment coupling had to be considered. The results showed that mechanical loading led to more intense metastable pits and a decrease in the repassivation ability of the 17-4PH MSS (Figure 10 and Figure 11), which logically promoted the formation of stable pits (Figure 8). Under mechanical loading, stable pitting was observed (Figure 8) at a potential in which the MSS was in the passive domain when no mechanical loading was applied. Such a result could be explained, at least partially, by considering the influence of mechanical loading on the passive film properties. The work of Vignal et al. [50] showed that, depending on the stress level, the properties of passive film formed on a 316L SS were modified. In particular, above 70% of the YS_0_._2_ value, microplasticity started to appear in some grains, and therefore, the film formed above those grains had a degraded chemical composition and protective properties as compared with the film formed in the absence of mechanical loading, in agreement with other works [53,54,55]. Li et al. [55] also showed that, for a 13Cr steel (alloy 410 or UNS S41000) exposed to 3.5% NaCl solution, above a certain imposed mechanical stress, E_corr_ and E_pit_ moved towards more negative potentials, due to the accumulation of Cl^-^ and H^+^ ions in confined areas. Other works, all about the FRDR model, have shown that the passive film could locally rupture under mechanical loading, thus, leading to an intensification of the anodic dissolution reactions of the underlying metal when it was brought into contact with the aggressive electrolyte, and of the reactions necessary for the repassivation of the metal [44,45,46,47]. Finally, Wang et al. [56] used a cellular automata model coupled with the finite element method to analyse the growth of metastable pits and their transition to stability of SS under mechanical stress; they clearly showed that the growth rate of metastable pits was higher under stress than that under no stress, which was in perfect agreement with our results. During the EAC tests performed in the present study, more intense current transients should stabilise and lead to stable corrosion pits. Indeed, during exposure to NaCl solution under mechanical loading, some grains should start to plasticise earlier than others; the passive film could then be degraded, and even fractured. This should result in the intensification of anodic dissolution reactions, and therefore, an increase in transient currents. It could be assumed that, due to the differences in mechanical properties between austenite and martensite [57], the degradation of the passive film properties should occur more rapidly above the austenite grains. In other words, the passive film formed above austenite was not only intrinsically less protective due to a lower Cr amount, but it was also more susceptible to degradation under mechanical loading. This should contribute to explain the higher susceptibility to EAC of the AM samples as compared with the CM samples.

Nevertheless, even though pitting was identified as a major process in the EAC susceptibility of 17-4PH MSS, the influence of hydrogen could not be disregarded. Protons produced by anodic reactions (formation of the passive film as well as pitting) and subsequent cation hydrolysis may lead to the formation of hydrogen. Hydrogen may contaminate the passive film, making it more susceptible to initiation and propagation of stable pits in potential ranges where the material was supposed to be passive. Once the pits had formed, they constituted a confined environment for the intensification of anodic dissolution processes, and thus, for the production of hydrogen. Then, the hydrogen could interact with the dislocations and be transported, leading to the formation of hydrogen-rich zones where corrosion processes are promoted, resulting in the establishment of a self-sustaining system. In other words, this meant that an autocatalytic process had to be considered. This scenario was in agreement with the work of Thomas et al. [44] who showed that, on the basis of potentiodynamic tests carried out on steels not charged with hydrogen and cathodically precharged with hydrogen, the corrosion potential moved towards more negative values while the corrosion current increased for steels precharged with hydrogen. These authors also showed that the anodic dissolution of the Fe matrix increased considerably for hydrogen precharged steels. They also noted that, due to its reducing power, hydrogen could reduce the passive film and modify its properties when it was absorbed in the first atomic layers of the matrix, in agreement with other works [58,59]. In the present work, the higher hydrogen content measured for MSS produced by AM as compared with MSS obtained by CM, for identical exposure conditions, therefore, would be consistent with a higher growth of stable pits for the AM samples than for the CM samples, and perhaps even with more intense metastable pits for the AM samples than for the CM samples. Furthermore, the influence of reversed austenite on hydrogen uptake was a major parameter, and the ratio austenite to martensite had to be considered to understand the susceptibility to EAC of the 17-4PH MSS. Indeed, it is well-known that the solubility limit of hydrogen in austenite is much higher than in martensite [60,61,62,63,64], which could contribute to explain that AM samples of MSS were more enriched in hydrogen after the EAC tests than the CM samples. Then, the influence of hydrogen on the mechanical properties should be considered. Here, the austenite to martensite ratio was also a crucial parameter. Indeed, we have shown, in previous work, significant differences in the relaxation properties of MSSs produced by AM and CM due to the higher amount of reversed austenite in AM samples [65]. Furthermore, hydrogen was shown to increase the mobility of dislocations in austenite. This could contribute to explain the significant decrease in YS_0_._2_ and flow stress values observed on the tensile curves plotted for the AM samples of MSS after the EAC tests (Figure 6). Finally, as previously mentioned, pit propagation was associated with the austenite grains. Hydrogen/dislocation interactions that occur inside the austenite grains might impact pit propagation, and thus, pit morphology and stress distribution at the bottom of the pits. The contribution of hydrogen to the EAC susceptibility of 17-4PH MSS will be evaluated in future work.

Regarding the current work observations, the question of whether or not to apply a heat treatment could arise. We showed in a previous work [32] that as-built AM samples had higher i_pass_ values, but more positive E_pit_ values as compared with AM samples after H900 heat treatment. This was explained by referring to the amount of NbC particles; indeed, the solution annealing at 1040 °C induced an increase in the amount of NbCs as compared with the as-built samples. We also showed that no difference was observed in the austenite amount between as-built and H900 AM samples, with an austenite amount equal to 12.5 ± 0.3% for the as-built AM samples [13]. Then, as explained before, one major effect of the solution heat treatment at 1040 °C was to release the internal stresses associated with the AM process [13]. This should have a significant effect on the pitting and EAC susceptibility of the AM samples. All these results were obtained for the same parameters of elaboration of the AM samples. However, as indicated in the introduction, the building parameters had a significant influence on the microstructure of AM samples. Therefore, those observations showed that there was an intricate coupling between the elaboration parameters, the microstructural features, and the corrosion (pitting and EAC) resistance. It was then difficult, with the present results, to conclude about the interest of the H900 heat treatment. Nevertheless, it was of major importance to keep in mind that, regarding the mechanical properties, a post-building heat treatment, such as H900 heat treatment, was required.

## 5. Conclusions

The influence of microstructure on the pitting and EAC susceptibility of 17-4PH MSS was evaluated in NaCl solution.

Based on reference to the literature data, it was shown that pit initiation was mainly controlled by NbC particles, whereas the pit propagation rate was increased when the amount of reversed austenite was higher.

The results clearly evidenced that pitting was a major process in the EAC mechanism with pits acting as preferential crack initiation sites. Therefore, both pit initiation and propagation significantly influenced the EAC process, and the previous microstructural features had to be controlled to master the EAC susceptibility of 17-4PH MSS.

The issue was made even more complex due to the fact that mechanical loading influenced the pit initiation and propagation steps, promoting both steps. The results showed that mechanical loading had a significant influence on pit stability, which was more marked for the L-PBF samples, leading to higher EAC susceptibility for the L-PBF MSS as compared with its conventional counterpart. Even if hydrogen uptake occurred during the EAC tests, the influence of hydrogen formed during the passivation and pitting processes on EAC susceptibility was not the focus of this study.

The major result was that 17-4PH MSS produced by AM was more susceptible to EAC when the building parameters led to a significant amount of reversed austenite. This means that AM processes should be developed and the building parameters need to be fixed in order to control the amount of reversed austenite, but also the density and size of NbC particles.

## Figures and Tables

**Table 1:** Chemical composition of the AM and CM samples (Fe balance, wt.%. The S amount was lower than 0.005 wt.% for both the AM and CM samples of MSS. The amounts of N and O were 310 ± 6 (140 ± 2) ppm and <2 (<2) ppm for the AM (CM) samples, respectively).

	C	Cr	Ni	Cu	Nb	Mo	Mn	Si	P
AM	0.028 ± 0.002	16.20 ± 0.19	4.08 ± 0.10	3.56 ± 0.11	0.27 ± 0.02	<0.020	0.32 ± 0.01	0.71 ± 0.03	0.007 ± 0.002
CM	0.036 ± 0.002	15.42 ± 0.19	4.49 ± 0.11	3.24 ± 0.10	0.26 ± 0.02	0.15 ± 0.01	0.39 ± 0.01	0.35 ± 0.02	0.016 ± 0.003

**Table 2:** Average values (5 tests) obtained for the different parameters that are characteristic of the metastable pits for both the AM and CM samples of MSS.

	Number	I_pit_ (µA)	T_g_ (s)	T_r_ (s)	I_pit_/T_g_ (µA s^−1^)
AM	32	0.92 ± 0.25	3.2 ± 0.8	0.3 ± 0.2	0.288
CM	135	0.12 ± 0.06	1.0 ± 0.4	0.2 ± 0.2	0.120

**Table 3:** Average values (4 tests, i.e., about 80 pits analysed) obtained for the different parameters that are characteristic of the stable pits for both the AM and CM samples of MSS. N = number of pits per cm^2^.

	N (cm^−2^)	D_ap_ (µm)	D (µm)	h (µm)	f
AM	12	280 ± 25	340 ± 27	162 ± 41	2.1
CM	15	120 ± 22	180 ± 32	147 ± 33	1.2

**Table 4:** Parameters that are characteristic of the mechanical properties and extracted from the tensile curves plotted during the EAC tests (Figure 6). Errors on E, YS_0_._2_, and UTS are indicated in the table (±).

	E (GPa) ± 5		YS_0_._2_ (MPa) ± 2		UTS (MPa) ± 2		ε_f_	
	AM	CM	AM	CM	AM	CM	AM	CM
Air	203	201	1289	1251	1407	1389	0.678	0.956
OCP	208	209	1278	1248	1395	1385	0.672	0.949
OCP + Pol	220	222	1150	1203	1227	1310	0.512	0.808

## Data Availability

The raw/processed data required to reproduce these findings cannot be shared at this time, as the data also form part of an ongoing study.
